# A *Post Hoc* Analysis of the CKD-FIX Study Analyzing the Association Between Metformin Usage and Estimated GFR Decline

**DOI:** 10.1016/j.ekir.2024.02.011

**Published:** 2024-02-09

**Authors:** Isabelle Kitty Stanley, Andrew J. Mallett, Andrea K. Viecelli, Carmel M. Hawley, Christine E. Staatz, David W. Johnson, Elasma Milanzi

**Affiliations:** 1 Faculty of Medicine, The University of Queensland, Brisbane, Australia; 2 Institute for Molecular Bioscience, The University of Queensland, Brisbane, Australia; 3 Department of Renal Medicine, Townsville Hospital & Health Service, Townsville, Australia; 4 College of Medicine and Dentistry, James Cook University, Townsville, Australia; 5 Australasian Kidney Trials Network, The University of Queensland, Herston, Australia; 6 Department of Kidney and Transplant Services, Princess Alexandra Hospital, Brisbane, Australia; 7 Translational Research Institute, Brisbane, Australia; 8 School of Pharmacy, The University of Queensland, Brisbane, Australia; 9 School of Population and Global Health, University of Melbourne, Melbourne, Australia

**Keywords:** chronic kidney disease, kidney function, metformin

## Introduction

Chronic kidney disease (CKD) poses an increasing burden on global health; however, there are few interventions available to manage the condition. It has been hypothesized that metformin, a commonly prescribed oral antidiabetic drug, may potentially be able to slow the progression of CKD.^1^ Metformin is the first line treatment for patients with type 2 diabetes mellitus (T2DM) due to its beneficial effects on blood glucose control and its tolerability.^1^ It primarily imparts its therapeutic actions through activation of adenosine monophosphate-activated protein kinase, which subsequently inactivates the molecular target of rapamycin and its postulated downstream fibrogenic effects on the kidney.^1^ Metformin therapy may therefore improve outcomes in CKD in addition to the benefits of improved blood glucose control.

Metformin is used extensively worldwide for the management of T2DM, it is readily available, affordable, and has a well-established safety profile.^2^ Its primary side effects of gastrointestinal disturbances and vitamin B_12_ deficiency are both easily managed. More serious adverse events, such as metformin-associated lactic acidosis, occur very rarely.^3^ Because metformin is predominantly excreted unchanged in the urine, the risk of accumulation and subsequent adverse events may be increased when kidney function is reduced.^3^ Metformin has therefore generally been avoided in the later stages of CKD, and data on therapy in this population are limited.

In 2020, the Australasian Kidney Trials Network published findings from the Controlled Trial of Slowing of Kidney Disease Progression from the Inhibition of Xanthine Oxidase (CKD-FIX).^4^ This randomized placebo-controlled trial enrolled 369 adult participants with stage 3 or 4 CKD at risk of disease progression. The trial randomized participants to receive either allopurinol or placebo over 2 years of follow-up.^4^ Diabetic kidney disease was the cause of kidney disease in 45% of the population. In this *post hoc* analysis, a subpopulation of patients from the CKD-FIX study, who had stage 3 CKD and T2DM, was used to compare changes in kidney function over time in those who were and were not taking metformin at baseline.

## Results

A total of 97 participants from the CKD-FIX population with stage 3 CKD and T2DM were included in this *post hoc* analysis. Fifty-one were taking metformin at baseline and 46 were not. Eighty participants in the subpopulation completed 104 weeks of followed up. Baseline characteristics of these participants are provided in Supplementary Table S1. Of note, the sample population included a higher proportion of male participants than female (72% male vs. 28% female), those taking metformin had a higher mean baseline estimated glomerular filtration rate (eGFR, 43.9 ml/min per 1.73 m^2^ vs. 35.9 ml/min per 1.73 m^2^) and were older (67 vs. 61 years) than those not taking metformin. Diabetic nephropathy was the primary cause of CKD in both treatment groups.

**Figure 1:**
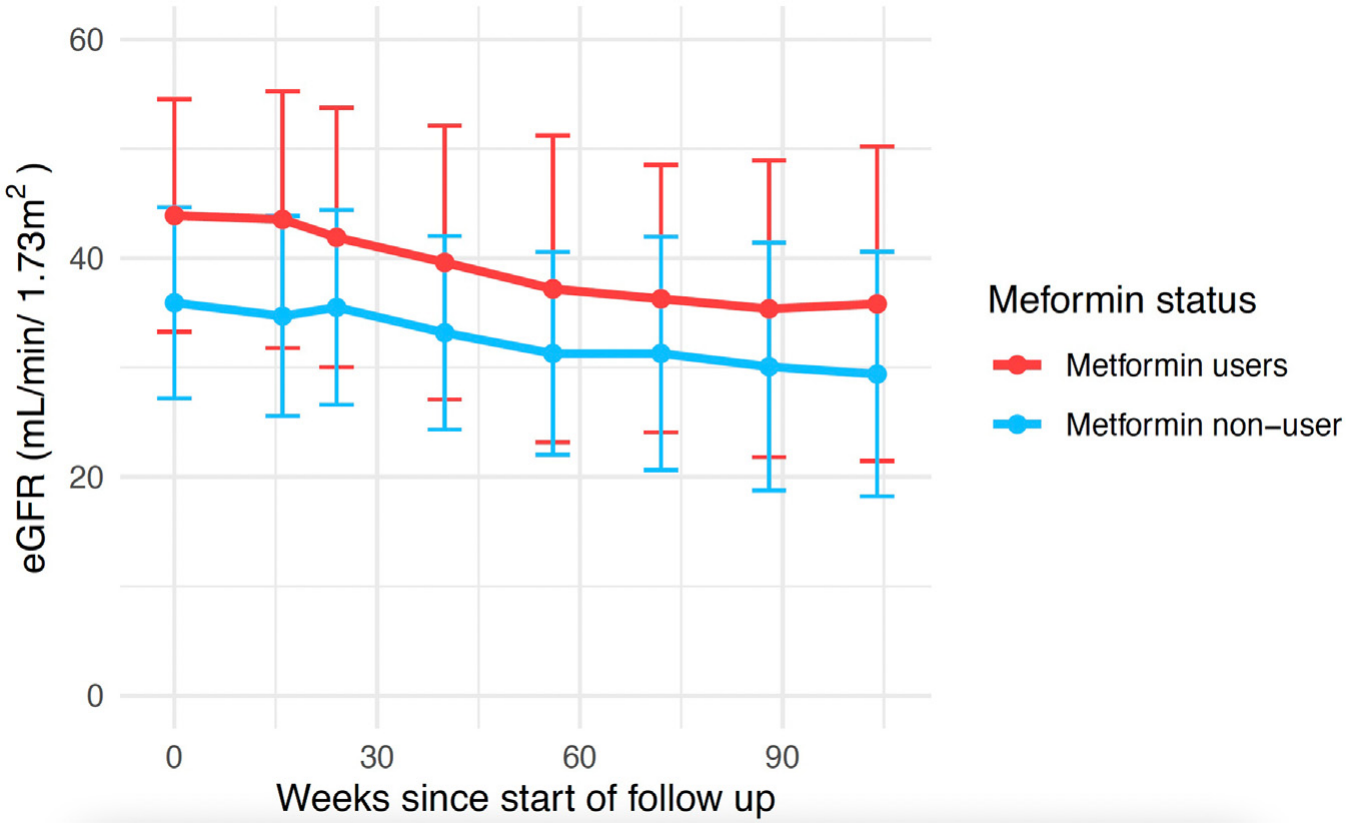
Mean eGFR (with SD) over time in participants included in post hoc analysis, by metformin status. eGFR, estimated glomerular filtration rate.

Mean eGFR values for those on metformin were higher at all time points (Table 1) and the individual participant trajectories were more variable than those not taking metformin (Figure 1). Trajectories were clustered using a growth mixtures approach, and a single pattern of decline was identified, implying that both groups had a similar trajectory. The between group difference in rate of change of eGFR was estimated using a linear mixed model and was not statistically significant (difference of 0.011 ml/min per 1.73 m^2^ per week [95% confidence interval, 0.043 to 0.022; *P* = 0.516]). Similarly, analysis of change in urine albumin-to-creatinine ratio showed no significant difference in patients treated with metformin compared to those who were not (Supplementary Figure S1).

**Table 1:** Mean eGFR±SD (mL/min per 1.73 m^2^) over time in participants included in *post hoc* analysis, by metformin status

Metformin status	Week since start of follow-up					
	Baseline	Week 16	Week 40	Week 56	Week 72	Week 104
Metformin users	43.9 ± 10.6	43.5 ± 11.7	39.6 ± 12.5	37.2 ± 14.0	36.3 ± 12.2	35.8 ± 14.4
Metformin nonuser	35.9 ± 8.7	34.7 ± 9.1	33.2 ± 8.8	31.3 ± 9.3	31.3 ± 10.7	29.4 ± 11.2

eGFR, estimated glomerular filtration rate.

Of the 51 participants treated with metformin, 18 (35%) and 7 (14%) experienced 30% and 40% declines in eGFR, respectively. Of the 46 participants not treated with metformin, 14 (30%) and 9 (20%) experienced 30% and 40% declines in eGFR, respectively. The between group differences were not significant.

The prevalence of serious adverse events and hospitalizations were similar between the 2 groups. In total, 50 participants reported at least 1 serious adverse event, 28 were participants who were prescribed metformin and 22 were not prescribed metformin. There was no significant difference between the 2 groups, in the incidence of serious adverse events or the incidence of hospitalization in this population. There were 4 participants who died, none of whom were being treated with metformin.

## Discussion

This analysis observed changes in kidney function measures in a subpopulation of participants from the CKD-FIX trial population, comparing those who were using metformin at baseline to those who were not; we included participants with T2DM and stage 3 CKD. At all time points, participants who were taking metformin had a higher eGFR than those who were not. Over the observational period, there was no significant difference in change in eGFR between the groups.

Given metformin’s primary indication for T2DM and its precautions in advanced kidney disease, participants without T2DM and with stage 4 CKD were excluded from this analysis. This created a population in which all participants had a diagnosis of T2DM and stage 3 CKD, facilitating fairer comparison of the effect of metformin in this population. Given the nature of this *post hoc* analysis and the potential for confounding factors to influence the results, the statistical approach of propensity scores (Supplementary Methods) was used to account for predetermined factors where possible. Furthermore, from the data available, adherence to metformin or cessation of therapy could not be assessed, which may have biased the results.

The small sample size (97 participants) included in this analysis limited the statistical power of the study to precisely detect between-group differences if they existed. Adequately powered clinical trials are required to answer this research question. An upcoming clinical trial of metformin in participants with CKD (NCT03831464)^5^ and an ongoing Cochrane systematic review will further investigate this topic.^6^

## Disclosure

DWJ has received consultancy/honorarium from Baxter Healthcare, Fresenius Medical Care, Astra Zeneca, AWAK, Ono, and Bayer; has received support for attending meetings and/or travel from Amgen and Ono; and received research funding for his institution from Baxter Healthcare, Fresenius Medical Care, and an Australian National Health and Medical Research Council Leadership Investigator Grant. AJM has received support for attending meetings and/or travel from Otsuka; participated on Advisory Boards without honorarium with GSK and MSD, and Board Member without payment with ANZSN council; and received investigator-initiated competitive research grants from National Health, Medical Research council of Australia, Medical Research Future Fund, Queensland Government, Australian Government, and Sanofi-Genzyme. CMH has received research funding for her institution from the National Health and Medical Research council of Australia and Medical Research Future Fund funding. All the other authors declared no competing interests.

## Supplementary Material

**Supplementary Methods.**

**Supplemental Reference.**

**Figure S1.** Mean urine albumin-to-creatinine ratio over time.

**Table S1.** Baseline characteristic of participants.
