# Supplementary material for: A Post Hoc Analysis of the CKD-FIX Study Analyzing the Association Between Metformin Usage and Estimated GFR Decline
Source: Kidney Int Rep. 2024 Feb 9;9(5):1525–7. doi: 10.1016/j.ekir.2024.02.011 (PMC11068973; doi:10.1016/j.ekir.2024.02.011)
Supplement: Supplementary File (PDF) [file mmc1.pdf]

Supplementary files

Supplemental methods

This study was a post-hoc analysis of data from the CKF-FIX clinical trial, which investigated the effect of allopurinol on rate of eGFR decline in patients with stage 3 or 4 CKD. A detailed description of the trial is available,[S1] but in brief, the study recruited adults with stage 3 or 4 CKD (eGFR, 15 to 59 mL/min/1.73m^2) deemed to be at increased risk of CKD progression. An increased risk of disease progression was defined as a urinary albumin: creatinine ratio of 265 mg/g or higher or a decrease in eGFR of at least 3.0 mL/min/1.73m^2 in the preceding 12 months. A subpopulation was formed for this analysis, applying additional exclusion criteria. All non-diabetic participants were excluded, in addition to those with stage 4 CKD.

Participants in this subpopulation were classified based on baseline use of metformin, as either metformin users or non-users. Confounding factors were accounted for using the statistical approach of propensity scores. Variables deemed to potentially confound the relationship between metformin exposure and eGFR change were randomised treatment group (allopurinol vs. placebo), albuminuria, CKD stage 3 group (A, eGFR >45mL/min/1.73m^2 vs. B, eGFR ≤45mL/min/1.73m^2), country (Australia vs. other), age, sex, body mass index, cause of kidney disease (diabetic nephropathy vs. other), serum phosphate levels, and ethnicity (Caucasian vs. other).

Descriptive statistics and visualisation were used to explore the data. This was followed by a clustering analysis of the individual trajectories to identify patterns of eGFR decline. A growth mixture approach[S2] was used and only one pattern of decline was identified. The linear mixed model was used to estimate the rate of change in eGFR (slope) difference and its corresponding confidence intervals. In conjunction with the p-values, these were used to test if the eGFR

slopes for the two exposure groups were different. The model was weighted by propensity scores (inverse probability weighting), which were estimated through a logistic regression of the metformin status and the potential confounders. Secondary aims was to assess if the proportion of participants achieving 30% and 40% declines in eGFR differed between those taking and those not taking metformin, and if the frequency of serious adverse events or mortality differed between groups. A chi-square test was used to assess any association. Analyses were conducted using R statistical software v4.2.1 (R Core Team, Vienna, Austria, 2020).

Supplemental References

S1. Badve S.V, Pascoe E.M, Tiku A, et al, Effects of Allopurinol on the Progression of Chronic Kidney Disease. *N Engl J Med*. 2020;382(26): 2504-2513. https://doi.org/10.1056/NEJMoa1915833

S2. Andruff H, Carraro N, Thompson A, et al. Latent Class Growth Modelling: A Tutorial. *Quant Meth Psych*. 2009;5(1):11-24. https://doi.org/10.20982/tqmp.05.1.p011

Supplemental Table S1: Baseline characteristic of participants included in post hoc analysis. Results presented as mean (SD) or number.

	**Metformin users N=51**	**Metformin non-users N=46**	**Overall N=97**	**p-value**
**Age**				0.02
Mean (SD)	66.9 (10.8)	61.3 (12.5)	64.3 (11.9)	
**Sex**				0.4
Male	35	35	70	
Female	16	11	27	
**eGFR (mL/min/1.73m^2)**				<0.001
Mean (SD)	43.9 (10.6)	35.9 (8.7)	40.1 (10.5)	
**Ethnicity**				0.4
White/Caucasian	32	37	69	
Other	19	9	28	
**Cause of kidney disease**				0.4
Diabetic nephropathy	39	34	73	
Hypertension/Vascular	5	9	14	
Reflux nephropathy	1	0	1	
Polycystic kidney disease	2	0	2	
Other	1	2	3	
Unknown	3	1	4	
**BMI (kg/m^2)**				0.6
Normal (19-24.9 kg/m^2)	3	3	6	
Overweight (25-29.9 kg/m^2)	10	13	23	
Obese (≥30 kg/m^2)	38	29	67	
Missing	0	1	1	
**Urine albumin-to-creatinine ratio (mg/g)**				0.8
Mean (SD)	151.5 (170.7)	166.0 (206.7)	158.3 (187.6)	
**Serum phosphate levels (mmol/L)**				0.4
Mean (SD)	1.18 (0.21)	1.21 (0.20)	1.19 (0.20)	

Supplemental figures:

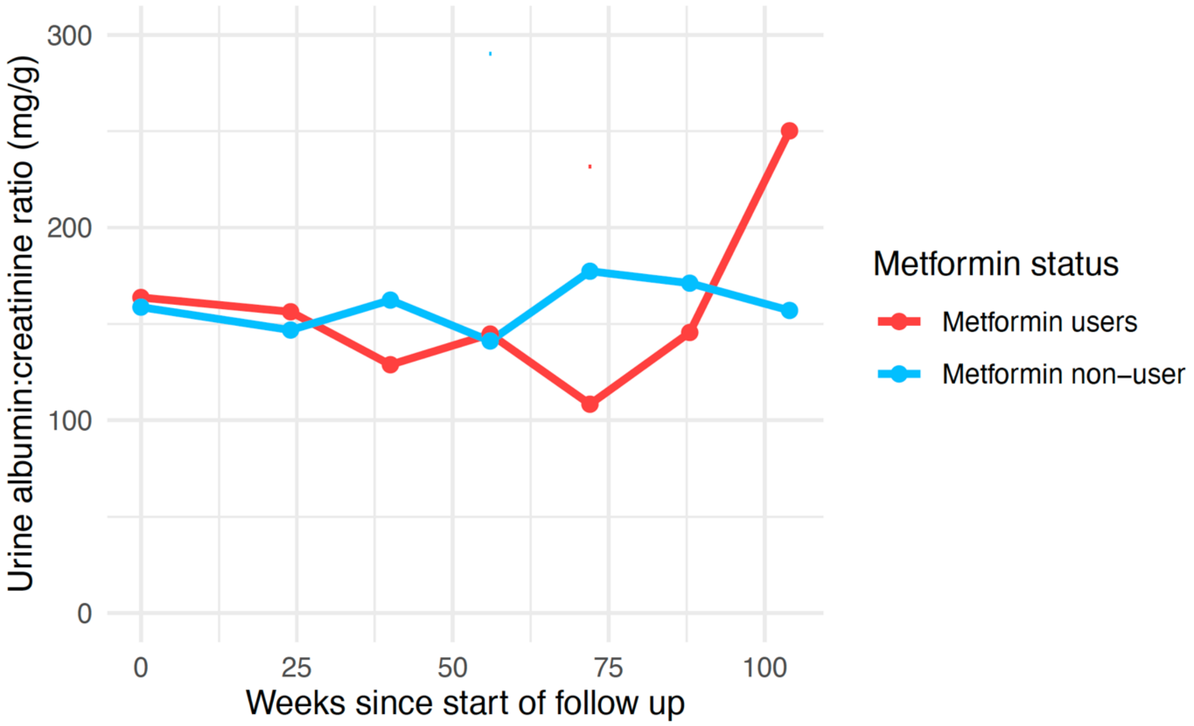

Supplemental Figure S1: Mean urine albumin:creatinine ratio (mg/g) over time in participants included in post hoc analysis by metformin status
